# The Ophthalmoscope in Medical Practice

**Published:** 1904-10-15

**Authors:** C. O. Hawthorne

**Affiliations:** Physician to the Central London Ophthalmic Hospital; Assistant Physician to the North-West London and Royal Waterloo Hospitals


### The Ophthalmoscope in Medical Practice.

By C. O. HAWTHORNE, M.D., M.R.C.P., Physician to the Central London Ophthalmic Hospital; Assistant Physician to the North-West London and Royal Waterloo Hospitals.

One of the most conspicuous features of modern medicine is the attention which is paid to the collection of objective evidences of disease as a basis for diagnosis. New modes of examination have greatly increased the opportunities of the physician in this direction. The methods of physical examination, chemical and bacteriological investigations, and the use of the microscope have revealed facts which have proved to be of the highest value in the art no less than in the science of medicine. Indeed, to neglect these facts nowadays is to court disaster. The older physicians, it is true, though without the aids that the modern schools possess, attained by their unceasing devotion to careful clinical observation great skill and shrewdness in arriving at correct conclusions from the subjective symptoms and general condition of the patient. And it may well be said that their example in these respects ought not to be neglected. But whilst such studies are not in any sense less essential now than they were in an earlier day, they can be considerably aided by more exact methods of investigation. The patient remains and always must remain the central point of clinical observation, but the interrogation of his condition is enlarged, more especially on its objective side. Among the means by which this extension of the study of the objective evidences of disease has been rendered possible the ophthalmoscope claims a position of much prominence. It has this very special value, that it allows the observer actually to see in the fundus oculi of the patient the peripheral termination of one of the cranial nerves and a complete scheme of distribution of an artery and vein as well as a number of other complex and highly organised tissues. The clinical information gained does not, as in the ordinary physical examination of the chest and abdomen, rest on an inference from certain observed physical facts. It is, on the contrary, direct and first-hand, for it depends on the immediate sense-perception of the observer. The facts of the fundus oculi are revealed as distinctly, for example, as the tint of the skin or the state of the tongue. Hence it is no cause for surprise that the ophthalmoscope has a large range of opportunity in the field of medical diagnosis. The nervous, vascular and other tissues which it displays to the eye of the physician are, of course, subject to the pathological changes common to the tissue groups to which they severally belong. But whilst these changes, more particularly in their earlier developments, are in other parts of the body appreciated for clinical purposes with difficulty and only indirectly by the symptoms or signs they produce, they are in the fundus oculi displayed for direct inspection. Such an arrangement suggests that evidences of disease in the nervous and vascular tissues may sometimes be detected before, in some instances long before, they can be appreciated in other parts of the body, and experience proves this to be the case. Again, the anatomical and physiological relationships existing between the various parts of the eyeball on the one hand and the brain and its related structures on the other has this result, namely, that various forms of intracranial disease are apt to produce, and sometimes at an early stage of their development, distinctive changes in parts that lie within the range of ophthalmoscopic examination. The ophthalmoscope is, therefore, much more than an instrument for the detection of local disease or injury in the tissues of the eyeball. Rather it gives to all who recognise the necessity for cultivating a wide outlook on the processes of disease an opportunity for the direct scrutiny of certain parts of the nervous and vascular systems and of detecting there, when they are present, changes that may mean either some generalised pathological condition, or localised disease within the skull or in some more remote part of the body. It is sufficient to state these considerations to show that for the purposes of modern medical diagnosis the ophthalmoscope is not less than a necessity.

Regarding the cultivation of the use of the ophthalmoscope for such purposes as have just been indicated, the best advice, even though it be dismissed as a counsel of perfection, is to use it for every patient. For this there are good and sufficient reasons. In the first place such a practice confers dexterity and makes the practitioner apt in the use of the instrument even in circumstances not favourable to its employment. Secondly, it has to be remembered that the fundus oculi does not present a picture uniform in all its details in every individual; not only are there differences in tint but there are also peculiarities of structure which yet do not transgress physiological limits and the range of which can only be appreciated by prolonged experience. In short, it is only those who have learned the extent of area covered by the normal, or the not-abnormal, who are in a position to recognise with confidence what is certainly and without question pathological; this being more particularly true of the slight changes which mark the early beginnings of the processes of disease. There is yet another reason for constant use of the ophthalmoscope. It is provided by the fact that the effects of disease, even in an extreme degree, may exist in the fundus oculi without any impairment of vision. Many years ago, for example, Dr. Hughlings Jackson taught the profession that optic neuritis, even severe optic neuritis, is consistent with a full measure of vision. A parallel statement may be made of other and not less serious changes in the fundus oculi, and hence if the ophthalmoscope is only used when the patient complains of visual defect some of its most useful opportunities will be frequently overlooked. For those who would be dextrous in the use of the ophthalmoscope, confident in the distinction between normal and abnormal ophthalmoscopic facts, and unfailing in the detection of the latter whenever they exist, there is only one safe rule and practice, and that is to examine each fundus oculi in every patient. It sounds formidable, but when cultivated as a matter of routine, it is, at the outside, an affair of a few minutes.

Another condition, valuable, though perhaps not essential, to successful ophthalmoscopic examination, is a practical knowledge of the errors of refraction to which the dioptric mechanism of the eyeball is liable. These errors in some instances, in many cases of myopia, for example, are associated with gross ophthalmoscopic changes, the significance of which can only be appreciated by those who recognise the defect and its relationship to the ophthalmoscopic facts. In other instances the existence of an error of refraction produces appearances in the fundus which more or less closely simulate the appearances met with in disease, and thus the observer who is not familiar with this relationship may be led into seriously erroneous conclusions. And yet again, it is only those who are accustomed to recognise the presence of refractive errors and to meet them by the use of appropriate lenses to whom is possible that sharply-defined view of the fundus necessary for the appreciation of fine changes, and for the confident interpretation of ambiguous ophthalmoscopic appearances. Hence, without claiming a thorough knowledge of the several errors of refraction as an absolute necessity to a successful use of the ophthalmoscope, it must be insisted on as a great advantage.

One more hint may be permitted, namely, that so far as possible the use of mydriatics should be avoided. There are exceptional cases in which the pupil is so small that an inspection of all parts of the fundus is impossible; but for the most part a quite satisfactory examination can be conducted without artificial aids. Not only are these unnecessary, but, in addition, they cause the patient inconvenience; their use also means a waste of time, and in elderly patients they carry a certain risk of glaucoma. Further, the practice is liable to be credited with the responsibility for any failure of sight which may subsequently occur. On all these grounds, therefore, mydriatics are best avoided. Certainly their use as a routine measure in ophthalmoscopic examinations is much to be condemned, though they may be necessary in occasional instances.

A single drop of a 2 per cent. solution of cocaine produces some degree of dilatation of the pupil, though for full dilatation homatropine may be necessary. Small discs or lamellæ containing both these drugs are prepared and are very convenient for carrying in the pocket or bag. Atropine should never be used for an ophthalmoscopic examination; the paralysis of accommodation it produces lasts for several days, whereas with homatropine the accommodation recovers in 24 hours or less. In adults, too, the use of atropine involves a risk of glaucoma.

Here it may be also mentioned that a dark room is by no means necessary for ophthalmoscopic examination. The patient may be readily examined when sitting up in bed, or indeed without assuming this position, and it is good practice to accustom oneself to use the ophthalmoscope with the patient in the recumbent posture. In cases of coma or severe illness this, obviously, may be the only available method. Drawing the blinds of the sick-room will give quite a sufficient degree of darkness, and the light for the illumination of the fundus can be obtained from a lamp or candle.[1] With this, a few mydriatic discs, and his ophthalmoscope, the practitioner is fully equipped for exploring the fundus oculi in almost every case which can come under his observation.

A full description of the ophthalmoscopic changes that may be met with in medical cases would form a considerable volume, and all that can be attempted in this article is to indicate some of the more common of these changes and to point out in what manner they severally aid the physician in reference to diagnosis and prognosis. It will, perhaps, be most convenient to refer to the facts to be observed by the ophthalmoscope in various diseases which are of somewhat frequent occurrence.

### Intracranial Tumour.

The most common condition in the fundus oculi seen in cases of intracranial tumour is double optic neuritis. Indeed, it may be fairly said that when this condition exists, and more especially when it exists in a severe form, the diagnosis of intracranial tumour is the first suggestion which occurs to the physician. When associated with severe headache and with vomiting or other obviously cerebral symptom, there can be little hesitation about the diagnosis. The neuritis in most cases is a comparatively late result of intracranial tumour, and hence merely confirms the suspicions based upon other symptoms. Yet its value in this respect is very considerable, for pain may of course result from many causes, and being a subjective experience of the patient, is not susceptible of exact estimate by the physician. Hence, when double optic neuritis is discovered, the case passes out of the region of probability into that of practical certainty. The diagnosis has for its support a fact which is entirely independent of the patient's personality, and it can therefore be presented in a correspondingly confident tone. In such circumstances, therefore, the ophthalmoscope performs a very welcome function, for it makes secure the diagnosis, and lends an equally sure note to the proposal for treatment. Unfortunately the existence of double optic neuritis does not serve to indicate either the size, nature, or position of the tumour, and therefore is of no value as a guide to the site suitable for surgical interference. But whilst the general statement is true that optic neuritis is rather a late than an early result of intracranial tumour the rule is one to which there are numerous exceptions. The neuritis may indeed be the first, and for some time the only symptomatic evidence of such a tumour. Further, there is a class of cases met with in young adults at or shortly after puberty in which the symptoms of cerebral disturbance are very slight and yet in which there is every reason to believe the underlying condition is one of tumour. In these patients there is often nothing more than an occasional headache, sometimes confined to the early morning, and perhaps then attended with an attack of vomiting, at other times interfering for a whole day with attendance at school or business. In the intervals between the headaches the patient appears to be perfectly well and for the most part both himself and his friends regard his symptoms as trivial and unimportant. Repeatedly one hears such patients assert that at no time have they lost a single day's attendance at school or work, and if they have taken a medical opinion this, if the ophthalmoscope has not been used, is apt also to treat the symptoms as of little moment. In the course of a few months, however, the patient becomes sensible that his sight is failing, and on examination

[1] One of the most convenient pocket lamps is the "three-wick candle," made by Mr. Hawkesley, of 357 Oxford Street, W.

it is found that each disc shows a condition of post-neuritic optic atrophy which, when once established, is apt to go on to absolute blindness. That is to say, that a patient who complained of nothing more than occasional headache with or without an attack of vomiting must have had at the same time a double optic neuritis without any thought in his own mind or in that of his friends that he was in any sense seriously ill. The use of the ophthalmoscope in this stage would at once have altered the whole aspect of the case, and would have protected the practitioner from a most unfortunate and disastrous mistake. Such an experience vividly illustrates the necessity for an examination of the fundus oculi, even though no complaint is made of defective vision. It is sometimes said that the ophthalmoscope should always be used in diseases in which retinal changes are known to occur. But this is to wait until the diagnosis is made, and then to explore the fundus oculi merely for confirmation or as a matter of interest. The right policy is to make the examination in every case, for it is only in this way that the full diagnostic value of the method can be obtained. In the cases to which allusion has just been made, neglect of the ophthalmoscope in what is regarded often as nothing more than a "bilious attack," means a failure to discover a double optic neuritis, and an entire misinterpretation of the significance of the symptoms. Thus headache, however slight and occasional, and attended or not with vomiting, is a most decided claim for the use of the ophthalmoscope. It is well also to say here that repeated examination, however slight the suspicion of cerebral disease, is more than advisable. In some instances the development of a double optic neuritis takes place with great rapidity. In others it is a very gradual process. Obviously the desirable thing is to detect the condition at the earliest possible moment. And this is just one of the circumstances which emphasise the wisdom of that constant use of the ophthalmoscope here advised. Occasionally one sees an optic disc with a hazy, clouded, more or less irregular margin in a person in perfectly good health, and repeated examination shows this suggestion of optic neuritis to be a condition which undergoes no change, and to be, in short, normal to the individual. But were such appearances to be noted in an optic disc which had previously been seen to be free from them their significance would be very different. Hence the wisdom of frequent examinations, as the development of a new fact under observation is manifestly an event of importance.

Allusion may now be made to another group of cases of intracranial tumour in which the symptoms may lead to mistake. In these cases the first symptoms are those of an epileptic fit, and such fits may occur over a period of months or years without any suspicion that the case is other than one of idiopathic epilepsy. Possibly, if a careful investigation is made, there may be facts which might be held to suggest caution, such, for example, as the absence of any family history of epilepsy, the sudden onset of the fits without obvious cause at a time of life when the beginning of idiopathic epilepsy is not common, the persistence of headache, etc., etc. But as a matter of fact these cases often are regarded as cases of idiopathic epilepsy, and, moreover, the diagnosis appears to receive support from some measure of improvement obtained by the use of bromides. Sooner or later, however, something occurs to suggest an ophthalmoscopic examination, and there is then discovered a double optic neuritis. This is a further illustration of the wide range over which the symptoms of intracranial tumour may extend and how large is the service which the ophthalmoscope renders to the diagnosis. The symptoms may be so slight in degree that to the uninitiated the suggestion of an intracranial tumour seems almost absurd, or they may assume such a form that some other diagnosis rather than tumour is suggested, yet the whole position becomes changed with the discovery of double optic neuritis. As a final illustration on this point may be quoted the case of a man who complained of the recent development of a peculiar difficulty in walking associated with a character of gait that suggested in the strongest possible fashion a wilful attempt to deceive. Under observation, however, the edges of the optic discs in the course of a few weeks became obscure, and gradually a severe double optic neuritis developed. The possibility of malingering or functional disease was thus removed, and as a matter of fact the patient shortly afterwards died from the growth of a cerebral tumour. Reviewing, therefore, the whole position it is manifest that the ophthalmoscope has a large range of opportunity in the diagnosis of tumour growths within the skull. Upon prognosis, on the other hand, it has but little influence, as the presence or absence of optic neuritis gives but little aid in this direction. Perhaps little more can be said than this, that the sudden appearance of optic neuritis in a case in which other symptoms of tumour have existed for some considerable time, is an event of ill omen; it generally means extension of the tumour, and an early fatal termination of the case.

### Meningitis.

As might be expected for anatomical reasons, it is chiefly in basal meningitis, rather than in meningitis spread over the convex surfaces of the hemispheres, that changes in the fundus oculi are to be observed. It is possible in basal meningitis that there may be a considerable swelling of the discs, but this is unusual. The more frequent condition is an increased redness of the discs, as these are watched from day to day, with gradual obscuring and swelling of the margins by inflammatory exudation. This is another illustration of the value of repeated examinations. Mere redness of the disc may be normal in the individual examined, but the development of hyperæmia as a new fact is of some moment. Here, too, comes in the value of practice with the refraction ophthalmoscope, as it is only by this method that the slighter degrees of swelling of the disc can be appreciated. It must, however, be allowed that in many cases of tubercular meningitis no change can be appreciated in the optic discs, whilst in others they appear so late that their only value is to confirm the diagnosis. But in a certain proportion of cases the ophthalmoscope is of great value. Every practitioner is familiar with cases of this disease in which the development of events is both prolonged and ambiguous, the state of the patient varying from day to day and no confident diagnosis being possible. In some of these the changes in the optic discs already described may be observed, and in such circumstances the value of the ophthalmoscope is very great. Another change in the fundus sometimes seen in tubercular meningitis is the appearance of one or more tubercles in the choroid—white or yellowish-white nodules, slightly swollen, looking about the size of a millet-seed, and with ill-defined edges. They are of considerable diagnostic value, and are seen in general tuberculosis as well as in tubercular meningitis. As both these conditions often present much clinical difficulty the discovery of tubercle in the choroid may be of great assistance. Between tubercular meningitis and simple posterior basic meningitis the ophthalmoscope will hardly help to a confident distinction. Both in the one and the other the optic discs may show no change, or they may be reddened and swollen as already described.

Renal Disease.

Perhaps albuminuric retinitis is one of the best known retinal changes recognised as significant in medical practice. Much care has been spent in the description of the white dots and patches which distinguish this condition and of their arrangement in stellar form in the macular region. The accuracy of this description is beyond question, but two remarks may be made on it. In the first place this form of retinitis may be very closely simulated if not exactly copied in glycosuria, and also exceptionally in intracranial tumour. In the second place many changes in the fundus oculi other than a "typical albuminuric retinitis" may result from renal disease and, therefore, to rely solely upon ophthalmoscopic appearances for purposes of diagnosis is as unsafe as it is unscientific. Optic neuritis, a diffuse œdema of the retina, retinal hæmorrhages, changes in the walls of the arteries, even choroiditis, may each be associated with kidney disease and, therefore, equally with retinitis, call for careful examination of the urine. The form of kidney disease in which albuminuric retinitis is most frequently seen is chronic interstitial nephritis, though it may also occur in other chronic renal changes. Its diagnostic value is specially marked in those exceptional cases where kidney disease exists without albuminuria, and in a given case it would have value in distinguishing organic from functional albuminuria. Though it is quite true that retinal changes are usually only found in an advanced stage of chronic kidney disease, it often happens that they are the first-discovered facts on which the diagnosis depends. Frequently it is the case that they cause some interference with vision and thus the patient who, perhaps, considers himself in excellent health in other respects, consults an ophthalmic surgeon, by whom the retinal disease is discovered. In other instances there is no interference with vision, but various other symptoms lead to an ophthalmoscopic examination and to subsequent examination of the urine. From what has been already said it is obvious that albuminuric retinitis means a bad prognosis and this is more particularly true in chronic interstitial nephritis. In the parenchymatous form of kidney disease the prognostic significance is not so great and occasionally even there may result much improvement in the retinal condition; this may also be observed in retinitis accompanying albuminuria in pregnancy. It must also be added that sometimes even in granular kidney retinal changes may both be noted early and may improve under appropriate treatment of the renal disease. Another ophthalmoscopic fact of great value in the diagnosis of chronic renal disease is evidence of degeneration in the walls of the retinal arteries—the so-called "silver-wire arteries." There is yet another group of cases of renal disease in which the diagnosis is very apt to be overlooked unless the fundus oculi is examined. The patients are for the most part young women; they do not complain of any disturbance of the functions of the kidney or bladder, and dropsy is entirely absent. Headache is a common symptom as is also loss of flesh and strength. Another symptom is failure of sight. Whether this is or is not present it is common to find evidences of neuritis or retinitis or of both, and hæmorrhages into the retina may also be observed. For the most part the patient does not regard herself as at all seriously ill. Yet the condition is a most anxious one. There is the most imminent danger of uræmic symptoms in a dangerous and severe form, and, indeed, the outlook in these cases is almost hopeless. This is obviously a very marked contrast to the superficial aspect of such a case when it first comes under observation as an example of headache or of some mistiness of vision, and the true nature of such a case may readily be missed unless the fundus oculi is examined. Lastly, in connection with renal disease may be mentioned the temporary loss of sight without ophthalmoscopic change which sometimes occurs in uræmia.

Diabetes Mellitus.

In this disease retinal changes are less common than in the case of renal disease. They occur in the form of hæmorrhages and also as white dots and patches not unlike those seen in albuminuric retinitis. There are certain differences between the appearances in the two diseases, but these are mainly a matter of ophthalmoscopic refinement. The ultimate diagnosis rests with an examination of the urine. Retinal changes in diabetes are usually amongst the later developments of the disease. But it is not always so, and occasionally what appears at first sight to be an example of chronic dyspepsia will on examination prove to be a case of glycosuria with retinal changes.

Tabes Dorsalis.

The ophthalmoscopic fact to be noted in tabes dorsalis is the white appearance of the disc which proclaims the existence of optic atrophy. To that statement may be attached the remark that mere whiteness of the disc is hardly sufficient in any case to justify a conclusion of optic atrophy, though to the expert eye there may be attendant features which carry conviction. But if the undue whiteness of the disc is seen to develop under observation, or if with it there is failure of visual acuity or contraction of the visual field, the diagnosis of atrophy is made secure. Of course, with well marked spinal evidences of locomotor ataxia the development of optic atrophy is hardly needed to strengthen the diagnosis. But it must be remembered that optic atrophy is sometimes the first symptom of the disease; and, indeed, it may be said that any patient with optic atrophy, not otherwise explained, is probably the subject of degenerative changes in his nervous system which will sooner or later produce ataxia or other symptoms of spinal disease. The chief practical value of the recognition of optic atrophy as a possible symptom of tabes dorsalis is found in those cases where the spinal symptoms are slight and ill developed. Thus a patient comes complaining of some pains in his lower limbs, or of a little difficulty in reference to the function of his bladder, the true significance of which only becomes evident if it is found that he has no knee-jerks, or that there are signs of commencing optic atrophy. The same experience may apply to more or less frequent attacks of gastric disorder, which under the searchlight of the ophthalmoscope may be found to be true gastric crises. In reference to optic atrophy a remark has to be made somewhat similar to that offered in connection with optic neuritis—namely, that its presence is quite consistent with good vision, so far, at least, as the patient's sensations are concerned. Central vision may remain entirely unaffected, even to an advanced stage of the atrophy, but the impression suggested by the appearance of the disc will be confirmed by the use of the perimeter, which will show a gradually increasing concentric contraction of the visual field. It is often the case that when optic atrophy is an early fact in the development of tabes dorsalis the spinal symptoms are much delayed, sometimes even for years, but this is not a uniform experience, and it may be found that a patient who apparently has no ground for complaint other than failure of sight will in the course of a few weeks or months lose his knee jerks and show other signs of sclerosis of the posterior columns of the spinal cord. In such an instance the early observation by the ophthalmoscope is obviously of great prognostic value.

### Disseminated Sclerosis.

In this disease, as is well known, the difficulty is to distinguish between organic and functional disturbance. The difficulty is all the greater as hysterical facts may be associated with the structural changes of organic disease. It is here then that the discovery of optic atrophy becomes of service and makes evident that whatever else there be in the case there is an underlying element of structural change. Disturbances of sight just as of other senses are common enough in hysteria but optic atrophy necessarily means something more than functional disturbance and cannot be due to hysteria.

In nervous diseases other than the above the ophthalmoscope has a not unimportant part to play, but in disseminated sclerosis and in tabes dorsalis it may exercise an almost commanding influence.

### Chlorosis.

Changes in the fundus oculi in chlorosis are not common, but they are found occasionally and may be of very great importance. The one most frequently met with is double optic neuritis and here, as in other instances, this may exist without any prejudicial effect on vision until post-neuritic optic atrophy is produced. Hence there is a claim for the examination of every case of chlorosis with the ophthalmoscope. If this is neglected a certain number of instances of double optic neuritis will be overlooked. Further, as a result, there may be less vigour in treatment than is advisable and thus a risk of optic atrophy and defective vision will be incurred. For under suitable measures the optic neuritis of chlorosis is an eminently curable affection. The other risk which is attached to optic neuritis in chlorosis is that its discovery may lead, and not altogether unnaturally, to the suspicion of some more serious condition than chlorosis, for example, cerebral tumour. This is all the more likely seeing that in both conditions headache may be a prominent symptom, and in both too there may be vomiting, as gastric complaints are not infrequent in anæmic women. Further, however it is to be explained, it is certain that the optic neuritis of chlorosis may be accompanied by an ocular paralysis, for example, of the sixth nerve, and this might well be quoted in the circumstances in support of the view that the patient was the subject of a coarse intracranial lesion. Headache, optic neuritis, and an ocular paralysis form indeed a combination strongly suggestive of organic disease within the skull. When they occur together the wise course probably is to suspect that such a diagnosis is necessary. At the same time, it must be remembered that all three symptoms have in more than one instance been noted in an anæmic girl, and have completely cleared away under the influence of rest and large doses of iron. The same remark may be made of optic neuritis when existing apart from an ocular paralysis or other suggestion of intracranial disease. Whilst remembering that an existing anæmia may be a possible explanation, this view should only be adopted in an individual case after every attempt has been made to exclude with confidence the more serious diagnosis, and after the effects of treatment have shown that the lighter view receives support from the therapeutic aspect. From all this it is evident that the use of the ophthalmoscope can hardly be said to be an aid in the diagnosis of chlorosis, where indeed aid is hardly needed; but the method does occasionally discover a condition of which it is necessary to take note as one of the possibilities of the disease, and the existence of which is a claim for both careful scrutiny of the diagnosis, and for increased vigour and thoroughness in treatment. Another change sometimes noted in the fundus oculi in cases of chlorosis is the presence of hæmorrhages. A recent writer has gone so far as to say that this condition is common. Certainly such an experience is out of harmony with the usual verdict, which would rather say that in simple chlorosis or anæmia the presence of hæmorrhages is a most unusual event, and their detection is enough in itself to give rise to the suspicion that some other element enters into the case.

### Pernicious Anæmia.

It is this disease which is suggested when, with the general evidences of anæmia, hæmorrhages are found in the retina. The fact is one of much diagnostic value, as such a condition is present in the great majority of cases. Most practitioners have at some time or other been puzzled by a more or less severe anæmia occurring without obvious cause, and attended, perhaps, with evidences of pyrexia. In not a few of these an ophthalmoscopic examination would have promptly indicated the diagnosis. Here again it is worthy of note that this condition of retinal hæmorrhage may exist without any subjective sense of impaired vision. Leucocythæmia and purpura are other blood diseases in which retinal hæmorrhages occur. The latter are sometimes also found in cases of ague.

Syphilis.

Valuable evidence of the activity of the syphilitic poison, perhaps at a long prior date, can often be obtained by the use of the ophthalmoscope. In the fundus oculi such evidence is, perhaps, most frequently seen in the shape of patches of choroidal atrophy, each surrounded by a border of dark pigment. These patches may be confined to the periphery of the fundus, and thus produce no sensible effect on the patient's vision. Less frequently there is a diffuse retinitis, perhaps attended with a fine dust-like pigmentation. Another result of syphilis is the presence of fine opacities in the vitreous. Apart from the fundus oculi, it often occurs that during ophthalmoscopic examination signs of a former iritis may be detected on the anterior surface of the lens capsule in the shape of adhesions and dots of pigment. Indeed, it is well always to look for such appearances by using a high plus lens in the ophthalmoscope; such traces of iritis may readily be overlooked in an examination of the eye, which is restricted to a mere superficial observation. It is not necessary to enlarge on the value of such evidence as is here indicated. The difficulty of obtaining a history of syphilis when many years have passed since the date of primary infection is a commonplace of practice, and anything which will help to a secure diagnosis in such a case is obviously of the first importance. The aid rendered by the ophthalmoscope in this direction is frequently of the highest service. Occasionally optic neuritis develops during the secondary stage of syphilis.

Inherited Syphilis.

Disseminated choroiditis is frequently seen in children the subjects of inherited syphilis, and it may in any individual be the only evidence of that disease. Provided, as is not infrequently the case, the macular region is not involved, there may be no complaint of defective vision. Retinitis and optic atrophy also occur in this disease, and with these vision is impaired. Another valuable sign can often be obtained by using a strong convex lens to examine the cornea, in which traces of vessels may prove that the patient had in former days suffered from an attack of interstitial keratitis.

It would be easily possible to produce many clinical records to substantiate the value of the ophthalmoscope to the physician in all of the above, and indeed also in many other diseases. Equally it would be possible to multiply the descriptions of changes in the fundus oculi which are of great clinical significance. But to attempt these ends would occupy much space, and is outside the purpose of this article. The aim here has rather been to offer a general sketch of the position which ophthalmoscopic changes may be made to occupy in the work of diagnosis for those whose practice involves attention to a great variety of the processes of disease. To regard examination of the fundus oculi as the province of the specialist, and to act in accordance with this creed, is voluntarily to exclude from the area of observation many facts which are of the highest clinical value and significance. That these facts are often independent of the sensations of the patient only makes them of more value to the physician. To cultivate the habit of observing them means at the outset a certain amount of effort and patience, but the early difficulties once overcome, there lies before the practitioner an extended scheme of clinical observation from which he will obtain almost constant assistance in his highly responsible work. The first contribution to this scheme is a knowledge of appearances within the limits of the normal. Upon this he will graft the power to appreciate pathological changes almost as readily as he trains his eye to distinguish the cutaneous evidences of the various eruptive fevers. Even if the out-patient department of an ophthalmic hospital is not at hand, observation of his own patients, aided by an atlas of plates representing diseased conditions of the fundus oculi, will, if he has the necessary determination, soon render his ophthalmoscope a valuable, indeed an essential, agent in his scheme of clinical work.

